# Cardiac-mimetic cell-culture system for direct cardiac reprogramming

**DOI:** 10.7150/thno.35574

**Published:** 2019-09-19

**Authors:** Seuk Young Song, Jin Yoo, Seokhyeong Go, Jihye Hong, Hee Su Sohn, Ju-Ro Lee, Mikyung Kang, Gun-Jae Jeong, Seungmi Ryu, Seung Hyun L. Kim, Nathaniel S. Hwang, Kookheon Char, Byung-Soo Kim

**Affiliations:** 1 School of Chemical and Biological Engineering, Seoul National University, Seoul, Republic of Korea; 2 Interdisciplinary Program for Bioengineering, Seoul National University, Seoul, Republic of Korea; 3 Institute of Chemical Processes, Seoul National University, Seoul, Republic of Korea

**Keywords:** cardiovascular disease, direct reprogramming, coculture, membrane, electric stimulation

## Abstract

**Rationale:** Cardiovascular diseases often cause substantial heart damage and even heart failure due to the limited regenerative capacity of adult cardiomyocytes. The direct cardiac reprogramming of fibroblasts could be a promising therapeutic option for these patients. Although exogenous transcriptional factors can induce direct cardiac reprogramming, the reprogramming efficiency is too low to be used clinically. Herein, we introduce a cardiac-mimetic cell-culture system that resembles the microenvironment in the heart and provides interactions with cardiomyocytes and electrical cues to the cultured fibroblasts for direct cardiac reprogramming.

**Methods:** Nano-thin and nano-porous membranes and heart like electric stimulus were used in the cardiac-mimetic cell-culture system. The human neonatal dermal fibroblasts containing cardiac transcription factors were plated on the membrane and cultured with the murine cardiomyocyte in the presence of the electric stimulus. The reprogramming efficiency was evaluated by qRT-PCR and immunocytochemistry.

**Results:** Nano-thin and nano-porous membranes in the culture system facilitated interactions between fibroblasts and cardiomyocytes in coculture. The cellular interactions and electric stimulation supplied by the culture system dramatically enhanced the cardiac reprogramming efficiency of cardiac-specific transcriptional factor-transfected fibroblasts.

**Conclusion:** The cardiac-mimetic culture system may serve as an effective tool for producing a feasible number of reprogrammed cardiomyocytes from fibroblasts.

## Introduction

Direct cardiac reprogramming of fibroblasts is an attractive therapeutic strategy for treating cardiovascular diseases because the adult heart has a low regeneration capacity 1. An immunologically identical cardiac tissue can be produced by direct reprogramming from the patients' tissue without the potential risk of tumorigenesis that occurs with induced pluripotent stem cells 2 and the ethical issue using embryonic stem cells. Previous studies have demonstrated that cardiac transcription factors, Gata4, Mef2c, and Tbx5 (GMT), can directly convert fibroblast to cardiomyocyte-like cells *in vitro* 1 and *in vivo* 3-5*.* Furthermore, several methods such as applying miRNAs 6, 7, small molecules 8-11, and environmental cues 12, 13 have been used to induce the direct cardiac reprogramming of fibroblasts.

Although *in vivo* direct cardiac reprogramming has shown positive effects on the *in situ* repair of the heart in diseased animal models 14, several obstacles remain for clinical translation. Determination of the optimum dosage of reprogramming factors is one hurdle 15. Furthermore, the *in vivo* viral delivery of reprogramming factors may not be safe for clinical translation. Therefore, the implantation of human cells reprogrammed *in vitro* represents an alternative strategy 16-19. However, cardiac reprogramming efficiency is lower *in vitro* than *in vivo* 4, 5, 20, 21. This suggests that the natural cardiac milieu may be a critical factor for direct cardiac reprogramming 2, 14, 22, 23, and delivery of cues in the cardiac microenvironment to fibroblasts may improve the efficiency of direct cardiac reprogramming* in vitro*.

**Figure 1:**
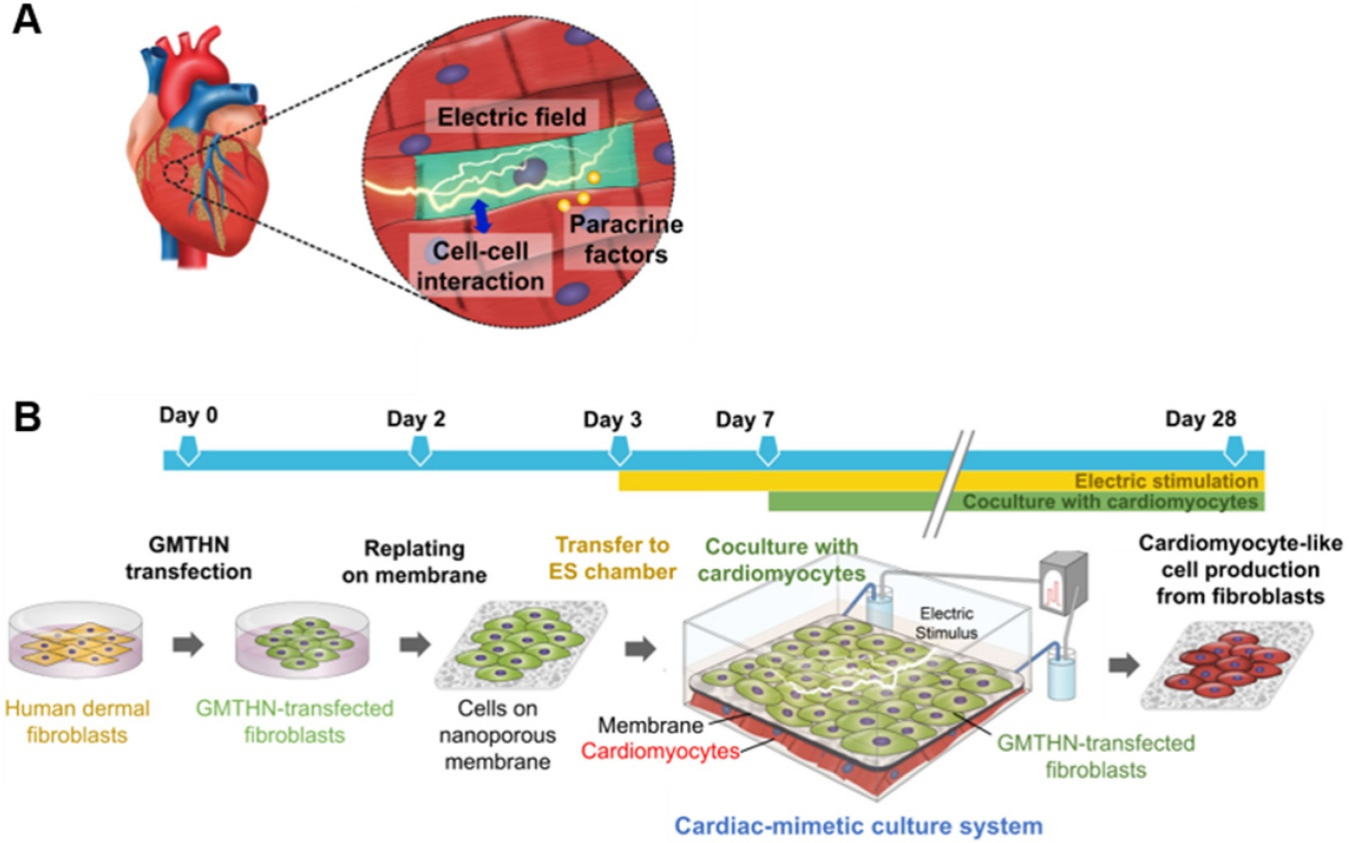
** Cardiac-mimetic culture system used to stimulate direct cardiac reprogramming in this study.** (A) Cues received by cardiomyocytes *in vivo* in the cardiac microenvironment. (B) A schematic diagram of the cardiac-mimetic culture system that mimics the cardiac microenvironment and provides interactions with cardiomyocytes and electrical cue to cultured fibroblasts for direct cardiac reprogramming.

In the heart, cardiomyocytes interact with neighboring cells through direct cell-cell interactions, various secreted cytokines, and electric signals (Figure 1A) 24. Through connexin 43, a channel protein in cardiomyocytes, cardiomyocytes directly engage in crosstalk and influence each other 25, 26. Previously, the mixed coculture of fibroblasts and cardiomyocytes in culture plates has been shown to promote the direct cardiac reprogramming of the fibroblasts *in vitro* 16. However, cell separation after coculture may not be feasible. Meanwhile, electrically active cardiomyocytes form an endogenous electric field in heart tissue, which is a distinctive characteristic of the heart. Previously, external electric stimulation (ES) has been applied *in vitro* to induce the cardiac differentiation of stem cells 27, 28. Moreover, external ES can pre-commit the dermal fibroblasts into the myocardial phenotype 29. No study to date, however, has applied external ES for direct cardiac reprogramming of fibroblasts *in vitro*.

Here, we introduce a cardiac-mimetic culture platform that can provide interaction with cardiomyocytes and electric cues to cardiac-specific transcription factor-transfected fibroblasts in order to promote direct cardiac reprogramming. The culture platform is equipped with a nano-thin and nano-porous membrane, which enables direct cell-cell contact interactions between cardiomyocytes and fibroblasts and paracrine factor interaction in coculture 30. Furthermore, the membrane makes cell separation feasible after coculture 30. The biodegradability and biocompatibility of poly(lactic-*co*-glycolic) acid (PLGA) allow the generation of readily implantable cardiac cell sheets without the need for the detachment and collection of the cultured cells from the membrane after direct reprogramming. Despite its degradability, the membrane maintains its structure and cell separation function during coculture for 3 weeks. We plated cardiac-specific transcription factor (Gata4, Mef2c, Tbx5, Hand2, and Nkx2.5; GMTHN) 31-transfected human neonatal dermal fibroblasts (HNDFs) on the membrane and cocultured them with murine cardiomyocytes in the presence of ES. After 4 weeks of culture, the ES and coculture promoted the expression of cardiac markers in HNDFs.

## Methods

### Preparation of PLGA membranes

For fabrication of nano-thin and nano-porous PLGA membranes, vapor-induced phase separation (VIPS) process was adopted during spin-coating. PLGA (LA:GA = 75:25, average molecular weight 66,000-107,000, Sigma Aldrich) was dissolved in tetrahydrofuran at 4 % (w/v) concentration. The silicon wafers were cleaned by piranha solution (a 7:3 mixture of H_2_SO_4_ and H_2_O_2_) for 20 min at room temperature. The PLGA solution was cast on wafers, and then spin-coated with 1000 rpm for 25 s in the closed chamber under a controlled relative humidity (RH) as proposed in previous studies 32-34. Since PLGA is not soluble in water, the porous characteristics of membrane change according to RH. For the membranes in this study, we set the RH to 45 ± 5 % 34. The edges of membranes were scratched and exposed to water, resulting in spontaneous detachment of membranes. Subsequently, the floating membrane was framed with the rectangular polyethylene terephthalate (PET) frame for easier manipulating.

### Characterization of membranes

The surface of PLGA membranes was analyzed by tapping-mode atomic force microscopy (AFM, Innova, Veeco, USA) and scanning electron microscopy (SEM, JSM-7600F, JEOL, Japan). The pore size of each membrane was investigated by AFM images using an image processing software, ImageJ (National Institutes of Mental Health, USA). The pores in the membranes were supposed to be the circle, and the pore diameter was calculated from the value of the area of each circle. Also, the membrane thickness was measured by AFM.

### Cell culture

HNDFs (Gibco BRL, USA; isolated from the skin of newborn male) were plated at a density of 2.5 x 10^3^ cells/cm^2^ and cultured in Medium 106 (Gibco BRL) supplemented with low serum growth supplement (Gibco BRL) at 37 °C in humid air with 5 %(v/v) CO_2_. The medium was replaced every other day until the culture is approximately 80 % confluent, and after that, exchanged every day. HL-1 cells (EMD Millipore, USA), murine cardiomyocyte cell line, were cultured in Claycomb medium (Sigma Aldrich) containing 10 %(v/v) fetal bovine serum (FBS; Gibco BRL), 0.1 mM norepinephrine (Sigma Aldrich), 2 mM L-glutamine (Gibco BRL), and 100 units/ml penicillin, and 100 μg/ml streptomycin (Gibco BRL). HL-1 cells were maintained at a high cell density to prevent dedifferentiate, so they were passaged at 1:3 split ratio only at 100% confluency. The culture plates for HL-1 cells were coated with 0.02 %(w/v) gelatin (Sigma Aldrich) and 5 μg/mL fibronectin (Sigma Aldrich).

### Cell viability on the membrane

HNDFs were seeded on PLGA membrane at a density of 1 × 10^5^ cells/cm^2^. The adhesion of HNDF on the membranes was imaged with a light microscope (IX71 inverted microscope, Olympus, Japan). After culture for various periods, live and dead cells were detected by live/dead assay with fluorescein diacetate (FDA, Sigma Aldrich) and ethidium bromide (EB, Sigma Aldrich).

### Cell-cell interactions between cocultured cells

HNDFs prelabeled with DiI (red, Sigma Aldrich) and HL-1 cells prelabeled with PKH67 (green, Sigma Aldrich) were seeded on membranes and gelatin/fibronectin-coated plates, respectively. At 24 h after cell seeding, HNDF-seeded membranes were transferred on HL-1 cell-seeded plate for coculture. After 48 h of coculture, the cocultured cells were captured using Z-stack imaging by a confocal microscope (LSM710, Carl Zeiss, Germany) and visualized with ZEN 2009 software (Carl Zeiss).

### The purity of collected HNDFs after coculture

HNDFs and HL-1 cells were plated on the membranes and gelatin/fibronectin-coated plates, respectively, for coculture. After 1, 2, and 3 weeks of coculture, we analyzed the expression of human-specific surface marker (human leukocyte antigen (HLA)) by flow cytometry. The HLA region is major histocompatibility complex (MHC) involved in the immune response in human, and is composed of three MHC proteins A, B, and C. These proteins exist on the surface of all nucleated human cells, thus anti-HLA-ABC antibodies specifically react with human cells. Therefore, it has been used for a xenograft marker which distinguishes human cells from the recipient cells in mouse xenograft models 35. In our coculture system, the human-specific marker (HLA) allows a distinction between human fibroblasts and murine cardiomyocytes. We confirmed the species-specific reactivity of anti-HLA antibodies using several human cells and non-human cells (Figure S2). The experimental procedure and results were described in Supporting Information in detail. For homogeneity analysis, HNDFs were collected by transferring the cell-attached membranes from the cocultures to 6-well plates. The cell-attached membranes were washed 3 times with phosphate-buffered saline (PBS, Biosesang, Korea). The cells were trypsinized and washed 3 times with cell staining buffer (Biolegend, USA). Subsequently, the samples were immunostained with fluorescein isothiocyanate (FITC)-conjugated anti-HLA antibodies (Biolegend) for 30 min on ice. After 3 times of washing by cell staining buffer, the expression of HLA was evaluated by flow cytometry (FACS Aria II, BD Biosciences, USA) installed at the National Center for Inter-university Research Facilities (NCIRF) at Seoul National University.

### Delivery of cardiac-specific transcription factors

For the construction of the plasmid, human Gata4, Mef2c, Tbx5, Hand2, Nkx2.5 (GMTHN, a gift from John Gearhart, Addgene plasmid # 46030) 31 were subcloned into an empty vector pBI-MCS-EGFP, a gift from Bert Vogelstein (Addgene plasmid # 16542) 36. HNDFs were transfected via electroporation (Neon^®^ Transfection System 100 μL kit, Invitrogen, USA) according to the manufacturer's instructions. Briefly, HNDFs were trypsinized and washed 3 times with PBS. The cells were suspended in a resuspension buffer to achieve the desired cell concentration of 1 × 10^7^ cells/mL. The cells and pDNA mixture were loaded into a 100 μL electroporation tip using NeonTM pipette and transfected with a single pulse of 1700 V for 20 ms according to the optimized protocols for primary cells (Invitrogen). After the pulse, the samples were transferred immediately to the dishes with the medium at a density of 3.5 × 10^4^ cells/cm^2^. The transfection efficiency was analyzed by flow cytometry (FACS Aria II, BD Biosciences) at 2 days after the electroporation. Also, the transfection is confirmed by RT-PCR analysis. Two micrograms of total RNA were used for cDNA synthesis with RT-PreMix (Bioneer, Korea). PCR was performed with PCR-PreMix (Bioneer) under standard PCR cycles consisted of an initial denaturation step at 94 °C for 5 min, followed by 35 amplification cycles consisting of 45 s of denaturation at 94 °C, 30 s of annealing at 62 °C, and 90 s of extension at 72 °C. Last, a final extension was performed at 72 °C for 10 min. PCR products were analyzed by UV irradiation on a 1.5% (w/v) agarose (Biosesang) gel stained with Redsafe^TM^ (Intron Biotechnology DR, Korea).

### Cardiac-mimetic culture

ES culture chambers were constructed as previously reported 37. Briefly, the culture chamber consisted of a Teflon block with channels, a Teflon lid, and a glass slide. Two rubber spacers positioned both sides of the glass slide as a cushion to prevent cracking of the glass and leaking of the culture medium. Salt bridges were prepared from flexible silicone tubing (1.5 mm in inner diameter, 3 mm in outer diameter, 15 cm in length, Korea Ace Scientific corp., Korea) filled with 2% (w/v) agarose solution in PBS and placed in the channel of the Teflon block. The salt bridges were immersed in PBS reservoirs with Ag/AgCl electrodes connected to a power generator. All components of the ES chamber were sterilized and assembled in a sterile hood.

Two days after transfection, GMTHN-transfected HNDFs were enzymatically detached and plated on the PLGA membrane at a density of 1 × 10^5^ cells/cm^2^. At 24 h after cell seeding, HNDF-attached membranes were transferred to the ES chamber, and the medium was replaced with low-glucose Dulbecco's modified Engle's medium (Gibco BRL) containing 20% (v/v) Medium 199 (Gibco BRL), 10% (v/v) FBS, and 100 units/ml penicillin, and 100 μg/ml streptomycin. ES of 1 V/cm was applied with a biphasic square pulse for 5 ms at 5 Hz frequency (Tektronix, USA) to mimic endogenous electrical signals in the heart 38. After 1 week of transfection, HNDF-attached membranes were stacked on HL-1 cells in the ES chamber for coculture. HL-1 cells were seeded on the gelatin/fibronectin-coated ES chamber at a concentration 10-fold higher than that of HNDFs, based on the previous coculture condition for the maturation of reprogrammed cells 16. The medium was replaced daily.

### mRNA quantification

Quantitative real-time polymerase chain reaction (qRT-PCR) was used to quantify the relative mRNA expression of samples. The total RNA was extracted (n = 3 per group) using TRIzol reagent (Invitrogen) and reverse-transcribed into cDNA (AccuPower® RT-PCR PreMix, Bioneer, Republic of Korea). The expression of cardiac and fibroblast markers (Table S1) was evaluated using the StepOnePlus real-time PCR system (Applied Biosystems, USA) with FAST SYBR Green PCR master mix (Enzynomics, Republic of Korea). Each gene expression was normalized by glyceraldehyde 3-phosphate dehydrogenase (GAPDH). The PCR consisted of 50 cycles of denaturing (95 °C, 10 s), annealing (60 °C, 15 s), and elongation (72 °C, 30 s). All the data were analyzed using the 2^-ΔΔCt^ method.

### Cardiac protein expression

Cell-attached membranes were transferred to 6-well plates (Corning), washed with PBS 5 times and trypsinized. For flow cytometry analysis, dissociated cells were fixed with 4% (w/v) paraformaldehyde (Biosesang) for 20 min, permeabilized with PBS supplemented with 0.15% (v/v) Triton X-100 (Sigma), and blocked with 10% (v/v) goat serum (Gibco) for 15 min at room temperature. Subsequently, cells were reacted with cardiac Troponin T (cTnT) antibody from mouse (1:200, Thermo Fisher, USA) at room temperature for 1 h. After washing with PBS, cells were incubated with the rhodamine-conjugated anti-mouse secondary antibody from goat (Jackson ImmunoResearch Laboratories, USA) at room temperature for 1 h. The cells were analyzed using FACS Aria II (BD Biosciences, USA) and flowing software ver 2.5.1. (Perttu Terho, Turku Centre for Biotechnology). For immunocytochemical staining, cells were fixed in 4% (w/v) paraformaldehyde solution for 20 min, permeabilized with PBS supplemented with 0.6% (v/v) Triton X-100 and blocked with 10% (v/v) goat serum for 2 h at room temperature. The samples were then reacted with cardiac Troponin T (cTnT) antibody from mouse (1:200) overnight at 4 °C. After washing with PBS, the cells were reacted with rhodamine-conjugated anti-mouse secondary antibody for 1 h at room temperature. The samples were mounted in 4,6-diamidino-2-phenylindole (DAPI, Vector Laboratories, USA) for nuclear staining. Fluorescence images were detected using a confocal laser scanning microscope (SP8 X, Leica, Germany).

### Immunoblotting

Western blot analysis was performed on the intracellular signaling molecules expressed in HNDFs cultured with the cardiac-mimetic culture system. The cell lysate was prepared with cell lysis buffer (Cell Signaling, USA) and underwent electrophoresis in a 10% (w/v) SDS-polyacrylamide gel. Protein bands were transferred to nitrocellulose membrane (Millipore Corp., USA) via the semi-dry transfer technique. The membranes were blocked with 5% (w/v) skimmed milk (Beck Becton Dickinson company, USA) solution for 2 h at RT, and subsequently incubated with antibodies against β-actin (1:3000, Abcam, USA), Akt, pAkt, p38 (1:1000, Cell Signaling Technology, USA), pp38 (1:1000, Abcam) overnight at 4 °C. The membranes were incubated with horseradish peroxidase-conjugated secondary antibody (Abcam) for 1 h at RT. The bands were developed using a chemiluminescence detection system (Amersham Bioscience, England).

### Statistical analysis

All quantitative data are expressed as a mean ± standard deviation. A one-way analysis of variance (ANOVA) with the Bonferroni test was performed to determine significant differences using GraphPad Prism (version 5.03 for Windows, GraphPad Software, San Diego California USA). A value of p <0.05 was considered statistically significant.

## Results and Discussion

### Nano-thin and nano-porous membrane

**Figure 2:**
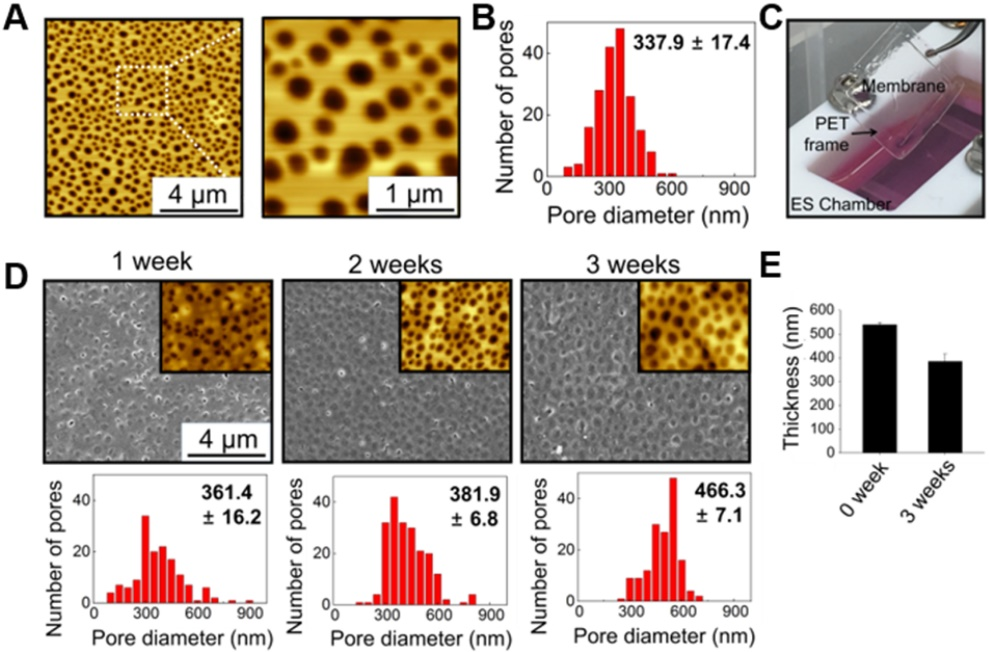
** Characterization of nano-thin and nano-porous PLGA membranes incubated for various periods in the culture medium in the culture system.** (A) AFM images and (B) pore size distribution of the membrane. (C) Photograph of the transferable membrane in the culture system. PET frame works as a support for easy handling of the membrane. Changes in (D) pore size and (E) thickness of the membrane during incubation in the culture medium in the culture system.

Figure 1 illustrates the culture system developed in this study, which can stimulate direct cardiac reprogramming by providing a cardiac-mimetic microenvironment. The culture system can provide interactions with cardiomyocytes and electric cue to fibroblasts. The system is equipped with a nano-thin, nanoporous, and the highly porous membrane, which can facilitate direct cardiomyocyte-fibroblast contact interaction and paracrine factor interaction in coculture. After coculture, cell separation is feasible by simply transferring the cell-attached membrane. We used PLGA for the membrane material because it is an FDA-approved, biocompatible material. Using the VIPS process, the well-defined nano-porous structure of the membrane was manufactured under closed conditions with constant RH (Figure 2A). In a coculture system, the membrane should not only serve as a barrier between the heterogeneous cell types but also facilitate communications in the cocultured cells. According to our previous study 33, the average pore size (338 nm) of the membranes used in this study (Figure 2B) is ideal for a coculture membrane.

The coculture membrane is installed in an ES chamber that provides external ES in the coculture environment. Since fibroblasts are cultured for 3 weeks to induce direct cardiac reprogramming, the coculture membrane should remain stable during this coculture period. We confirmed that the PLGA membranes retained their transferable features even after 3 weeks of coculture (Figure 2C). Moreover, after 3 weeks of incubation in aqueous conditions with ES, the porous structure was preserved well, as shown in AFM and SEM images (Figure 2D). The membranes were initially 538 nm thick with a pore size of 337.9 ± 17.4 nm. After 3 weeks of incubation with ES, the PLGA membranes slowly biodegraded, and the thickness and pore size changed to 385 nm and 466.3 ± 7.1 nm, respectively (Figure 2E).

### Suitability of the membrane for coculture

**Figure 3:**
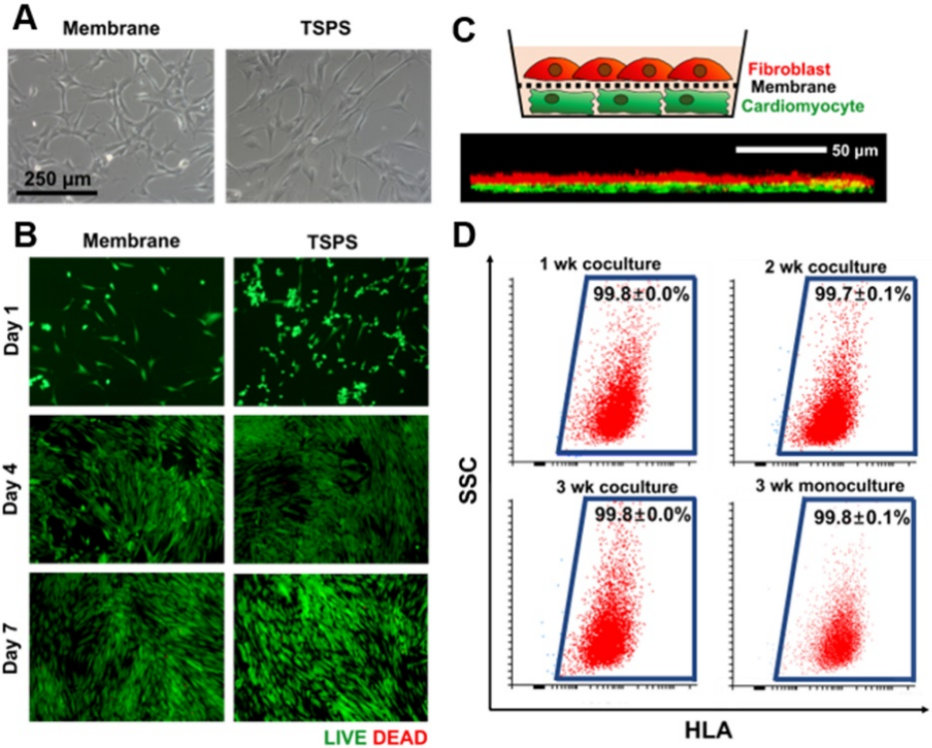
** Suitability of the membrane for cellular coculture.** (A) Microscopic images of HNDFs on the membrane and TSPS. (B) Viability of HNDFs cultured on membrane and TSPS at various culture time points as evaluated by Live/Dead assay. (C) Schematic illustration of coculture of HNDFs (red) and cardiomyocytes (green) using the nano-thin and nano-porous membrane and a z-stacked confocal image showing direct contacts (yellow) between HNDFs and cardiomyocytes through the membrane. (D) Homogeneity of HNDFs collected after 1, 2, and 3 weeks of coculture with murine cardiomyocytes, as evaluated by flow cytometry using FITC-conjugated anti-HLA antibodies. n=3 per group.

A coculture membrane should be biocompatible. To evaluate the biocompatibility of the PLGA membrane, HNDFs were cultured on the membrane and tissue culture polystyrene (TCPS) dishes, respectively. HNDFs cultured on the membranes showed the normal fibroblast morphology 24 h after seeding (Figure 3A). The live/dead cell assay at various time points confirmed that HNDFs could be cultured on the membranes with high viability, which was similar to that of cells cultured on TCPS (Figure 3B). Next, we confirmed the direct cell-cell interaction in the coculture system. HNDFs and cardiomyocytes were pre-labeled with a red and green fluorescent dye, respectively. After 48 h of coculture, the confocal microscopic image revealed that HNDFs and cardiomyocytes came into direct contact (yellow signal in Figure 3C) despite the presence of the membrane owing to its nanoscale thickness and high porosity (35.3±2.2 %, Fig S1). Finally, the homogeneity of the collected cells post-coculture was evaluated. For cell therapy, a coculture system must allow for the homogeneous cell collection post-coculture. HNDFs after 3 weeks of coculture were easily collected by the transfer of the cell-attached membranes. The cells can be implanted along with the biodegradable PLGA membrane because PLGA degrades several months following implantation 39. Moreover, the enzymatic collection of cells from a culture surface can reduce cell viability 33. The homogeneity of HNDFs separated after coculture was evaluated quantitatively by flow cytometry using a human-specific surface marker, HLA-ABC (Figure 3D). The results indicated a high homogeneity (>99.6%). This shows that the membranes act as a physical barrier to allow the homogeneous harvest of the cells after coculture. Collectively, the nano-thin and nano-porous PLGA membrane is suitable for coculture systems.

### Direct cardiac reprogramming by GMTHN

**Figure 4:**
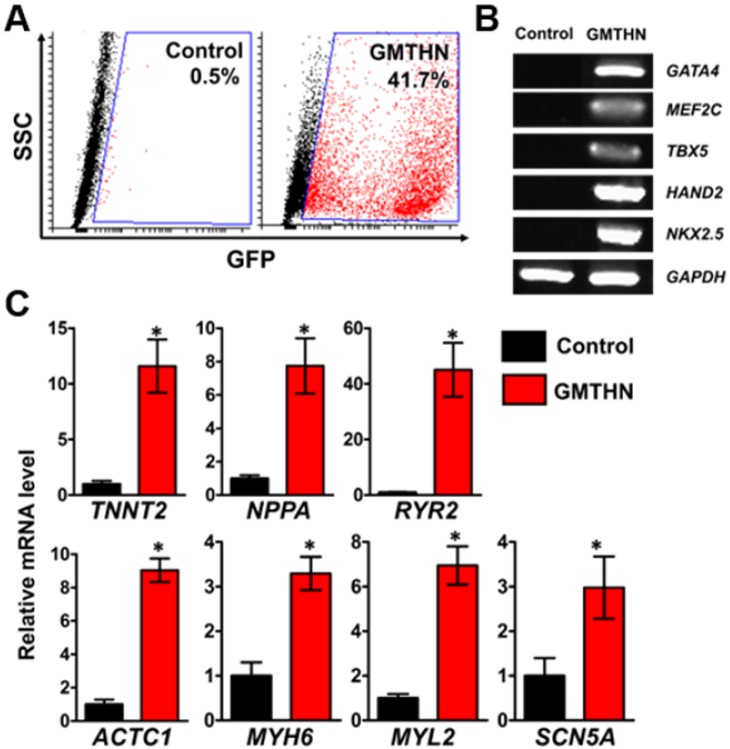
** Direct cardiac reprogramming of HNDF by delivery of cardiac transcription factors, GMTHN.** (A) GMTHN transfection efficiency (GFP-positive cell portion) of HNDFs as analyzed via flow cytometry after 2 days. (B) RT-PCR analysis of cardiac transcription factors. (C) mRNA expressions of cardiac markers in HNDFs after GMTHN transfection. The control indicates no transfection. * p < 0.05, n = 3 per group.

For direct cardiac reprogramming, a combination of cardiac transcriptional factors, Gata4, Mef2c, Tbx5, Hand2, and Nkx2.5 (GMTHN), was delivered to HNDFs using electroporation. It was previously reported that GMTHN generates induced cardiomyocytes 31. Forty-eight hours after the transfection, GMTHN-transfected HNDFs (GMTHN-HNDFs) were collected and analyzed by flow cytometry (Figure 4A) and RT-PCR (Figure 4B). GFP-positive (i.e., GMTHN-transfected) cells accounted for 41.7%, which is comparable to previously reported electroporation rate for neuronal reprogramming plasmids 40. On day 7, we evaluated the induction of cardiomyocyte-specific genes (TNNT2, NPPA, and RYR2) in HNDFs by qRT-PCR (Figure 4C) based on previous studies 16, 17. GMTHN-HNDFs showed higher expression levels of cardiac markers than empty vector-transfected HNDFs (the control group) 19. Moreover, the GMTHN transfection also upregulated the expression of cardiac genes related to sarcomeric structure (ACTC1, MYH6, and MYL2) and ion channels (SCN5A) compared to levels in the control group. These data show that the GMTHN transfection successfully induced cardiac markers in HNDFs.

### Enhanced direct cardiac reprogramming through cardiac-mimetic culture

To provide a cardiac-mimetic microenvironment for reprogramming, we replated GMTHN-HNDFs on nano-porous PLGA membranes 2 days after electroporation. On day 3, the GMTHN-HNDF-attached membranes were transferred to a custom-made ES chamber 37 to supply a biphasic square pulsatile ES that mimics endogenous electrical signals in the heart 38, 41. Based on previous studies on ES of cardiomyocytes and fibroblasts 42, 43, ES was initiated 24 h after cell attachment and maintained for the total culture time (Figure 1B). On day 7, coculture was initiated by transferring the GMTHN-HNDF-attached membranes to the cardiomyocyte-plated ES chamber.

**Figure 6:**
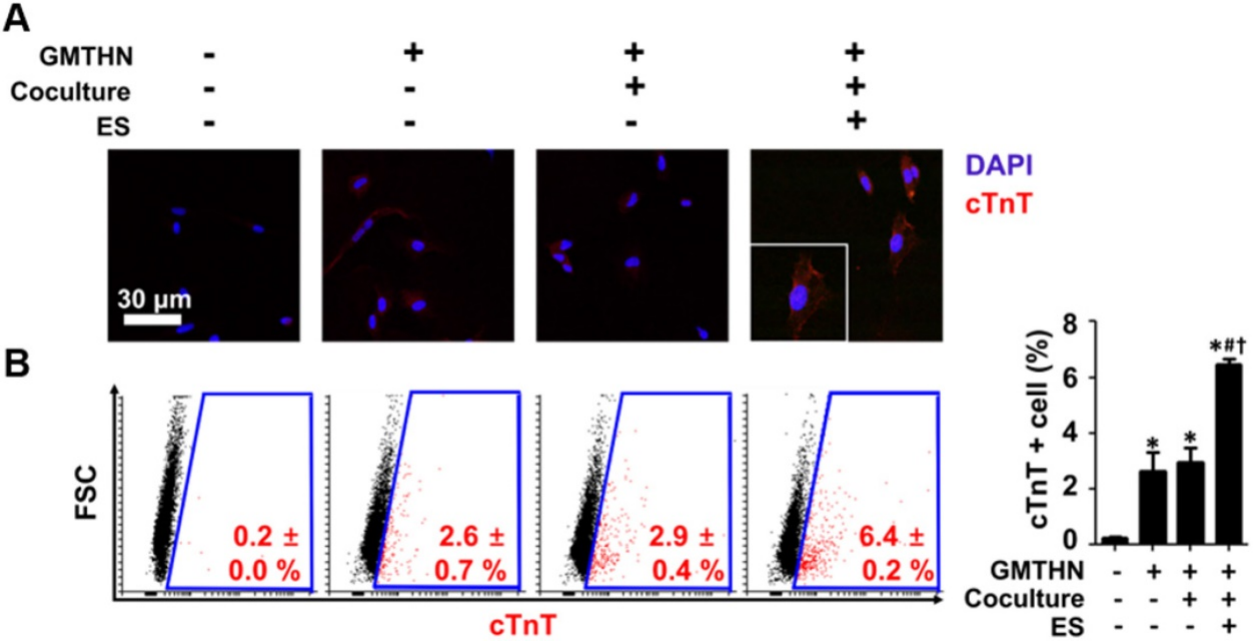
** Enhanced direct cardiac reprogramming in protein level by coculture and ES.** (A) Immunocytochemical staining for cTnT. (B) Flow cytometry analysis of cTnT-positive cells. *p < 0.05 versus group A, ^#^p < 0.05 versus group B, ^†^p < 0.05 versus group D. n=5 in group A, n=3 in group B, D, F.

The genes encoding cardiac contractile proteins (TNNT2 and MYL2), channel proteins (RYR2), cardiac peptides (NPPA), cardiac transcription factors (SIK1 and NFATC2), and a protein involved in cardiac metabolism (PHKA1) were upregulated significantly in the group F compared to the other groups. The TNNT2 and RYR2 genes were upregulated in group D, but the expression levels were higher in group F, implying that ES enhanced the direct cardiac reprogramming in the coculture environment. In contrast, COL1A2, a fibroblast marker, was downregulated in groups C, D, E, and F compared to that in the control groups A and B. Despite the lack of GMTHN, groups C and E showed higher cardiac marker expression levels than the control groups A and B, but the differences were not statistically significant. Therefore, we then evaluated the protein expression levels of cardiac markers only in groups D and F and the control groups A and B (Figure 6).

The protein expressions of cardiac markers were evaluated qualitatively and quantitatively using immunocytochemistry and flow cytometry, respectively (Figure 6). Cardiac troponin T (cTnT) has been used as an essential cardiac marker in previous studies. 16, 17 Similar to the gene expression data, the immunocytochemistry results showed that group F had the highest cTnT protein expression level (Figure 6A). Quantitatively, the most cTnT-positive cells were found in group F (6.4 ± 0.2 %), followed by group D (2.9 ± 0.4 %) (Figure 6B). The cells in group A merely expressed cTnT protein. These data indicate that GMTHN is essential for direct cardiac reprogramming. Interestingly, the percentage of cTnT-positive cells was not statistically different between groups B and D. This indicates that coculture alone is not sufficient to stimulate direct cardiac reprogramming. Collectively, the cardiac-mimetic culture system that supplies interactions with cardiomyocytes and electrical cues can improve the direct cardiac reprogramming.

We compared specific gene markers of mesoderm progenitor (SMAD3, HOXB2), cardiac mesoderm (GSC, MESP1), and cardiac progenitor / cardiomyocyte (KCNH2, SERCA2A, CACNA1C, MYH6, SCN5A, GJA1) 17, 44, 45 among the reprogrammed cells, human mesenchymal stem cells (HMSC) and human adult cardiomyocytes (HCM) using qRT-PCR analysis (Figure S4). The markers of mesoderm progenitor and cardiac mesoderm, highly expressed in a cardiogenesis, were negligibly expressed in the reprogrammed cells compared to MSC (Figure S5). The expression levels of the reprogrammed cells were not significantly different from those of HCM. In case of cardiac progenitor/cardiomyocyte markers, the mRNA levels were similar or even higher than those of HCM, in agreement with the previous studies in which directly reprogrammed cells 17, the late-stage ESC-CM and iPSC-CM 45 were compared to the human adult heart. The development stage-specific gene marker analysis suggests that the reprogrammed cells are cardiac progenitor cells or cardiomyocytes. Unfortunately, we could not observe any beating cells during the entire culture period. Thus the reprogrammed cells might not be mature cardiomyocytes.

After the direct reprogramming, we incubated the reprogrammed cells in stimulus-free culture for 4 weeks and analyzed the expression of the cardiac gene (Figure S5). Most of the cardiac gene markers dropped in a few weeks except for SIK1. This trend of decrease in cardiac marker expression over time is in agreement with *in vitro* culture of primary cardiomyocytes 46, 47. Despite the steep decline, their expression levels still surpassed statistically the level in the fibroblast group.

### Intracellular signaling related to coculture and ES

**Figure 7:**
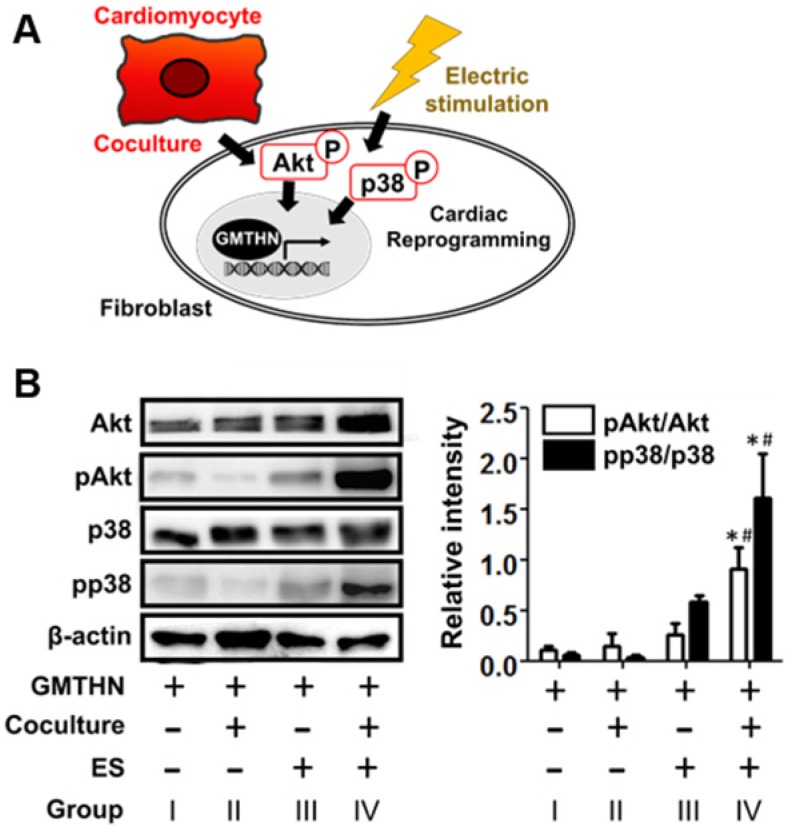
**Cardiac reprogramming-related intracellular signaling induced by interactions with cardiomyocytes and electrical cue in the cardiac-mimetic culture system.** (A) A schematic diagram of intracellular signaling involved in cardiac reprogramming and induced by interactions with cardiomyocytes and electrical cue. (B) Western blot analysis of GMTHN-transfected HNDFs cultured with or without coculture and/or ES for 7 days. *p < 0.05 versus monoculture without ES, ^#^p < 0.05 versus coculture without ES. n = 3 per group.

Next, we studied how cardiac-mimetic culture can stimulate direct cardiac reprogramming (Figure 7A). Though the full pathway in the cardiac direct reprogramming is still elusive, the phosphoinositol 3-kinase/Akt (PI3K/Akt) pathway 48, 49 and p38 mitogen-activated protein kinase (p38MAPK) pathway 49 were reported to serve as mediators of direct cardiac reprogramming in previous studies. The coculture with cardiomyocytes and ES (group IV) upregulated phosphorylation of Akt and p38 compared to the control group (group I), while coculture or ES alone did not (Figure 7B). Cardiomyocytes are known to secrete fibroblast growth factor 2 and vascular endothelial growth factor (VEGF) that promote direct cardiac reprogramming via the PI3K/Akt and p38MAPK pathways 49. ES stimulates VEGF expression in cardiomyocytes 50 for coculture and the phosphorylation of Akt and p38 in various cells 51-56 including fibroblasts 55 and ventricular myocytes 56 by itself. Together, the coculture with cardiomyocytes and ES stimulate direct cardiac reprogramming in our cardiac-mimetic culture system.

## Conclusion

We developed a cardiac-mimetic culture system that provides interactions with cardiomyocytes and electrical cues for direct cardiac reprogramming. The culture system is equipped with nano-thin and nano-porous membranes that allow direct cell-cell interactions between fibroblasts and cardiomyocytes in coculture and easy cell separation after coculture. After the delivery of the cardiac transcription factors, the coculture and ES supplied by the cardiac-mimetic culture system enhanced human direct cardiac reprogramming at both the gene and protein levels. The reprogrammed cells showed several gene markers of cardiac progenitor cells and cardiomyocytes comparable with human adult cardiomyocyte, but no contractile function. The coculture with cardiomyocyte and ES stimulated the direct reprogramming via PI3K/Akt and p38/MAPK pathway. The cardiac-mimetic culture system may be useful to produce a feasible number of reprogrammed cardiomyocytes from fibroblasts for cell therapy to treat cardiovascular diseases.

## Supporting Information

Quantitative real-time polymerase chain reaction(qRT-PCR) primer sequences and membrane image adjustment for analysis of pore size and porosity.

## Figures and Tables

**Figure 5:**
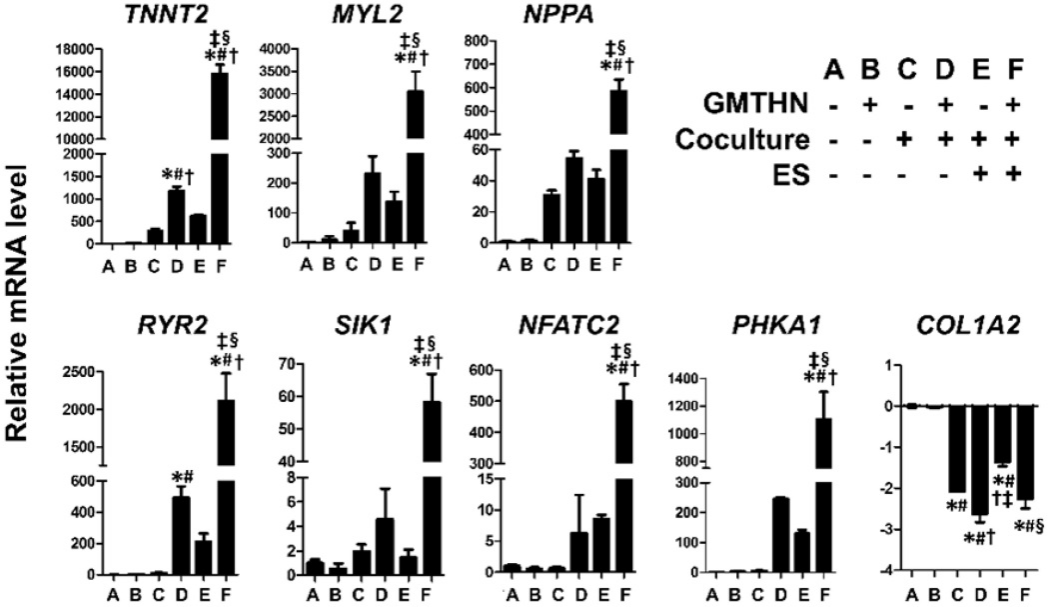
** Enhanced direct cardiac reprogramming in mRNA level by coculture and ES.** mRNA levels of cardiac contractile proteins (TNNT2, MYL2), channel proteins (RYR2), cardiac peptides (NPPA), cardiac transcription factors (SIK1, NFATC2), a protein involved in cardiac metabolism (PHKA1) and fibroblast marker (COL1A2). n=3 per group. *p < 0.05 versus group A, ^#^p < 0.05 versus group B, ^†^p < 0.05 versus group C, ^‡^p < 0.05 versus group D, ^§^p < 0.05 versus group E.

## Abbreviation

ES: electric stimulation
PLGA: poly(lactic-*co*-glycolic) acid
HNDF: human neonatal dermal fibroblast
VIPS: vapor-induced phase separation
PET: polyethylene terephthalate
AFM: atomic force microscopy
SEM: scanning electron microscopy
FBS: fetal bovine serum
PBS: phosphate-buffered saline
qRT-PCR: quantitative real-time polymerase chain reaction
GAPDH: glyceraldehyde 3-phosphate dehydrogenase
cTnT: cardiac troponin T
Akt: serine/threonine-specific protein kinase
