# Supplementary material for: Cardiac-mimetic cell-culture system for direct cardiac reprogramming
Source: Theranostics. 2019 Sep 19;9(23):6734–44. doi: 10.7150/thno.35574 (PMC6815967; doi:10.7150/thno.35574)
Supplement: Supplementary file 1 — Supplementary figures and tables. [file thnov09p6734s1.pdf]

Supporting Information

Cardiac-mimetic cell-culture system for direct cardiac reprogramming

Seuk Young Song[1], Jin Yoo[1], Seokhyeong Go[2], Jihye Hong[2], Hee Su Sohn[1], Ju-ro Lee[1], Mikyung Kang[2], Gun-Jae Jeong[1], Seungmi Ryu[2], Seung Hyun L. Kim[2], Nathaniel S. Hwang[1,2], Kookheon Char[1], and Byung-Soo Kim[1,2,3,*]

[1]School of Chemical and Biological Engineering, Seoul National University, Seoul, Republic of Korea

[2]Interdisciplinary Program for Bioengineering, Seoul National University, Seoul, Republic of Korea

[3]Institute of Chemical Processes, Seoul National University, Seoul, Republic of Korea

***Author to whom the correspondence should be addressed:**

Byung-Soo Kim, Ph.D.

E-mail: byungskim@snu.ac.kr

Tel.: +82-2-880-1509

Fax: +82-2-888-1604

Demonstration of the species-specificity of anti-HLA antibodies for cell homogeneity analysis

To evaluate human-specific reactivity of anti-HLA antibodies, three kinds of human cells (human neonatal dermal fibroblast (HNDF), human dermal fibroblast (HDF), and human mesenchymal stem cell (HMSC)) and four kinds of non-human cells (HL-1, mouse cardiomyocyte; H9C2, rat cardiac myoblast; B16F10, mouse skin melanoma; and L-929, mouse fibroblast) were plated on 6-well plates at a density of 3×10^4 cells/cm^2. We used high-glucose Dulbecco′s Modified Eagle′s Medium (DMEM-high) supplemented with 10% (v/v) FBS and 100 units/ml penicillin, and 100 μg/ml streptomycin except for HNDF and HL-1. After 2 days, the cells were trypsinized and washed by cell staining buffer (Biolegend) twice. The samples were resuspended in 50 μL of cell staining buffer and reacted with fluorescein isothiocyanate (FITC)-conjugated anti-HLA antibody (Biolegend, 1:10) on ice for 30 min. After washing twice, the cells were analyzed by flow cytometry (FACS Aria II, BD Biosciences). The anti-HLA antibody successfully reacted with all human cells (positive cells > 99.3%) but rarely reacted with non-human cells (positive cells < 0.6%) (Figure S2).

Stability of the cardiac markers after the direct reprogramming *via* cardiac-mimetic culture

After the direct cardiac reprogramming *via* cardiac-mimetic culture, the cell-attached membranes in group F were transferred into 35-mm petri dishes and washed 5 times by serum-free low-glucose DMEM (DMEM-low). The cells were incubated in 2 mL of DMEM-low containing 20% (v/v) Medium 199, 10% (v/v) FBS, and 100 units/ml penicillin, and 100 μg/ml streptomycin at 37 °C in humid air with 5% (v/v) CO_2 for 2 and 4 weeks. The medium was

replaced every other day. After the incubation, the total RNA was extracted and analyzed by qRT-PCR as previously described in the Method section.

Table S1. Quantitative real-time polymerase chain reaction(qRT-PCR) primer sequences

Gene	Direction	Sequence
GAPDH	Forward	TGC ACC ACC AAC TGC TTA GC
	Reverse	GGC ATG GAC TGT GGT CAT GAG
TNNT2	Forward	GAC AGA GCG GAA AAG TGG GA
	Reverse	CCT TCC TCC TCT CAG CCA GA
NPPA	Forward	AGC AAG CAG TGG ATT GCT CC
	Reverse	CTG GTC TGA CCT AGG AGC TG
RYR2	Forward	TGC TCA TTC TTT CAG CCA CCT
	Reverse	TCC GTG TTT TGT GAT GCA ACT
ACTC1	Forward	CAG ACC AGG ACT TGC AAC CT
	Reverse	TGC TCA GGG TGT CAA AGC AA
MYH6	Forward	AGG TCA ACA AGC TTC GAG CC
	Reverse	TTG GCA AGA GTG AGG TTC CC
MYL2	Forward	CTC ATC TCT CTC CCC CGA GT
	Reverse	CGG CCA CGA AGT ACC CAT AG
SCN5A	Forward	CAC TTT CCT TCG GGA CCC AT
	Reverse	CTG AGA CAT TGC CCA GGT CC
SIK1	Forward	CAG GGG CGG CCT GAG
	Reverse	CTC TGC TGC TCT AGC ACC TC
NFATC2	Forward	GGC ACG CGG TAG AGA AGA C
	Reverse	AGC CTC TCT CTG CAT CTG GA
PHKA1	Forward	ATG CTC GCT GTG GTG ATG AA
	Reverse	AAC CGA TCT AGC CCT CCC TT
COL1A2	Forward	AGG GGT CTC CAT GGT GAG TT
	Reverse	CCT CGG CTT CCA ATA GGA CC
SMAD3	Forward	AAC ATG TGG GCA AGA GCC G
	Reverse	GTG GGA ATG TCG CAT CCT GT
HOXB2	Forward	TGA ATT TGA GAG GGA GAT TGG GT
	Reverse	AAT GTC TCC AAG ACA GCG GG
GSC	Forward	CTC TGA TGA GGA CCG CTT CTG
	Reverse	GAG GAG AAA GTG GAG GTC TGG TT
MESP1	Forward	TGT GAG CAC CGA GGC TTT TT
	Reverse	TCC TGC TTG CCT CAA AGT GT
KCNH2	Forward	GAG CGC AAA GTG GAA ATC
	Reverse	GTT CTT CAC GGG CAC CAC AT
SERCA2A	Forward	GCA ATC TTT GAA TCC CCA TAC C
	Reverse	GCT GTT GAG GGC GTT ACA CA
CACNA1C	Forward	AAG GCT ACC TGG ATT GGA TCA C
	Reverse	GCC ACG TTT TCG GTG TTG AC
MYH6	Forward	AGG TCA ACA AGC TTC GAG CC
	Reverse	TTG GCA AGA GTG AGG TTC CC
SCN5A	Forward	ATT CTG GCT CGA GGC TTC TG
	Reverse	GAC ATT GCC CAG GTC CAC AA
GJA1	Forward	ACT GGC GAC AGA AAC AAT TCT TC
	Reverse	TTC TGC ACT GTA ATT AGC CCA GTT

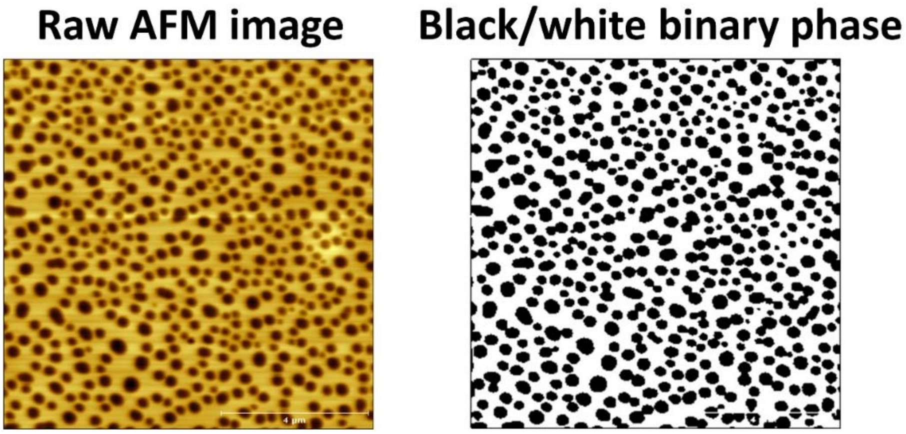

Figure S1. Membrane image adjustment for analysis of pore size and porosity. The raw AFM image of each membrane was adjusted to binary phase. Pore diameters were calculated from the area of black circles by Image J program. The ratio of the black area to the total area was considered as the porosity of the membrane.

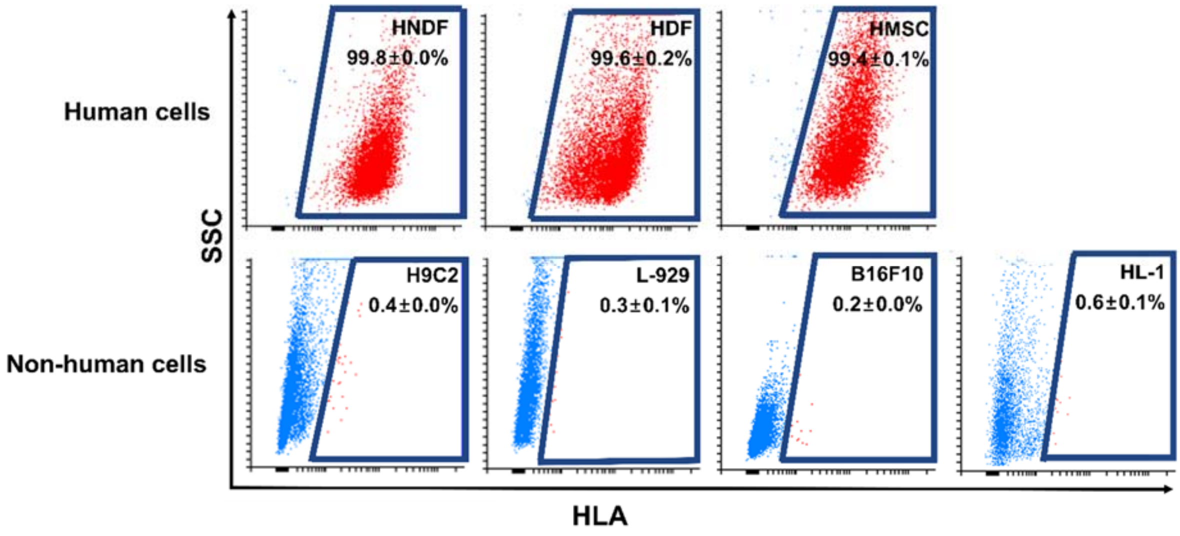

Figure S2. The species-specific reactivity of FITC-conjugated anti-HLA antibody. The red and blue dots are FITC-positive and -negative cells, respectively. The FITC-positive area of each cell line is determined by the unstained control cells. n=3 per group.

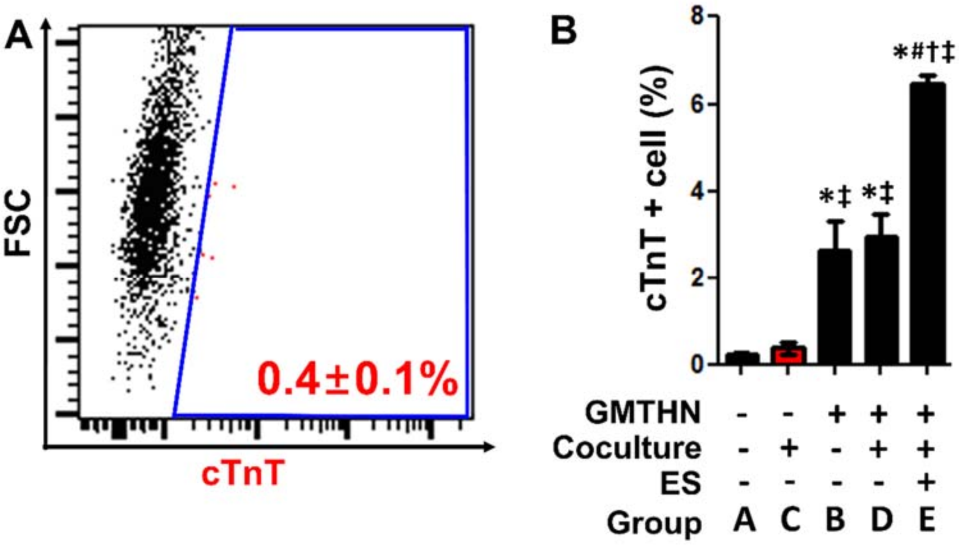

Figure S3. (A) Flow cytometry analysis of group C for cTnT-positive cells. n=3 per group. (B) Quantitative comparison with other groups. *p < 0.05 versus group A, #p < 0.05 versus group B, ‡p < 0.05 versus group C, †p < 0.05 versus group D.

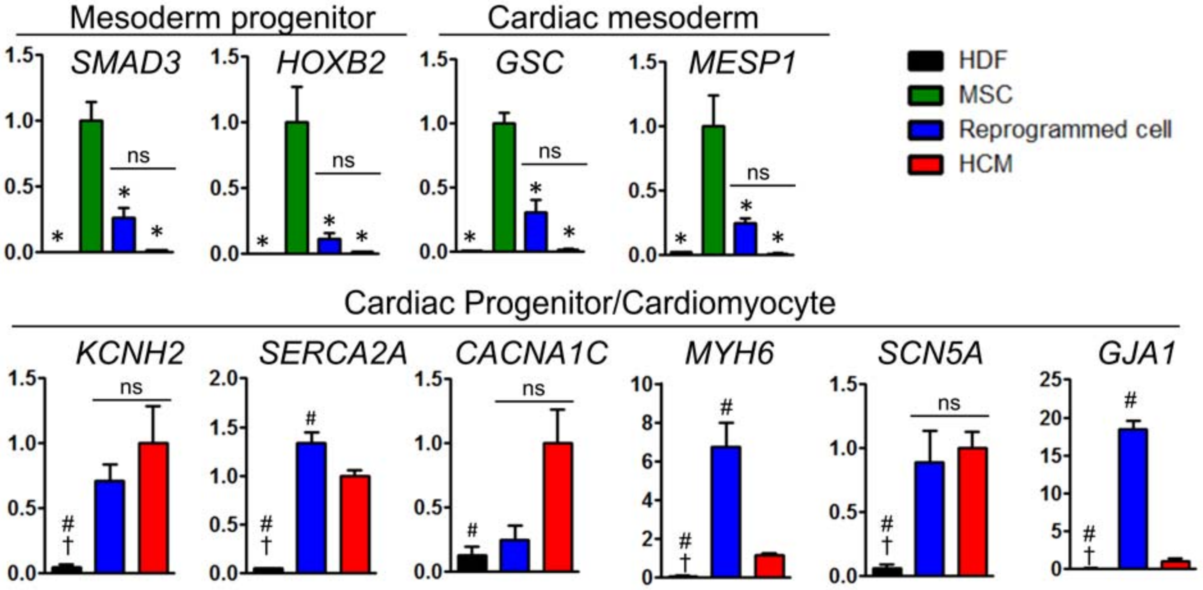

Figure S4. Expression of gene markers of mesoderm progenitor, cardiac mesoderm, cardiac progenitor, and immature cardiomyocyte. HDF, human dermal fibroblast; MSC, human mesenchymal stem cells, HCM, human cardiomyocyte; *p < 0.05 versus MSC, #p < 0.05 versus HCM, †p < 0.05 versus reprogrammed cells, ns, not significant. n=3 per group.

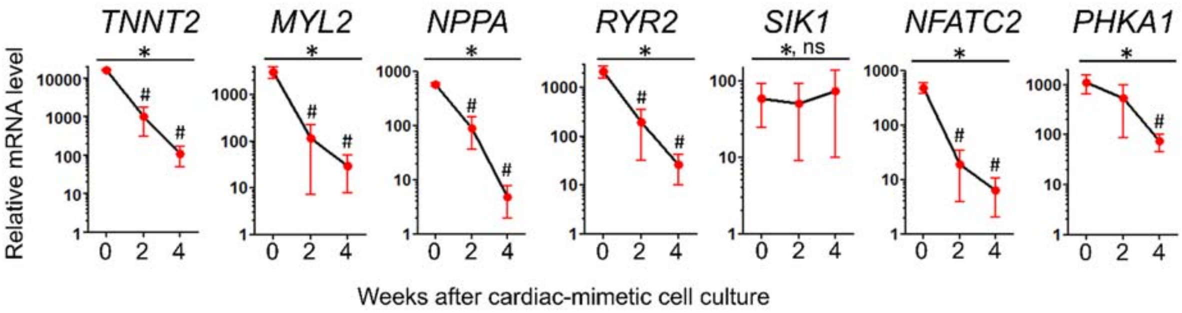

Figure S5. The expression profile of cardiac gene markers in stimulus-culture after reprogramming in group F. The expression levels of cardiac markers were normalized by the control fibroblast group (value=1) and presented in the log scale. *$p < 0.05$ versus control fibroblast, #$p < 0.05$ versus the initial value (0 week), ns, not significant, n=3 per group.
